# The Effect of Chronotype on Oppositional Behaviour and Psychomotor Agitation of School-Age Children: A Cross-Sectional Study

**DOI:** 10.3390/ijerph192013233

**Published:** 2022-10-14

**Authors:** Sandra Figueiredo, Rayane Vieira

**Affiliations:** 1 Department of Psychology of Universidade Autónoma de Lisboa, Coordinator Researcher in Psychology Research Centre (CIP) and of Foundation for Science and Technology, 1169-023 Lisbon, Portugal; 2 Department of Psychology, Universidade Autónoma de Lisboa, 1169-023 Lisbon, Portugal

**Keywords:** chronotype, health, oppositional behaviour, psychomotor behaviour, child development

## Abstract

The aim of this study is to examine the relationship between chronotype, classroom behaviour and school performance in 140 healthy school-age children attending various levels of education during the 1st cycle during 2021 in Portugal. In this cross-sectional and quantitative study, the Chronotype Questionnaire for Children (to assess the chronotype) and the Conners Scale—a reduced version was presented to the teachers (to assess behaviours such as excessive movement, inattention and oppositional behaviours)—were used. The methodology of this study followed a comparative method since the independent variables were not controlled, and therefore, it was still possible to compare the differences between the morning and evening groups. Statistical methods were used such as multivariate analyses, inter-item correlations and reliability tests, and descriptive tests were used for the percentile analysis. The sample was divided into three groups based on the identification of the chronotype—morning, intermediate and evening types—to further study the relationship between these chronotypes, their academic performance and classroom behaviour were studied. A multivariate analysis of variance revealed that there was a higher rate of oppositional behaviour in the morning type and no differences in the school performance during the two semesters (covering all of the school periods) regarding the chronotype effect, even with the analysis of regression parameters and covariates. On the other hand, the morning-type children showed a greater amount of motor agitation and impulsivity after controlling for the gender covariate. Age had an effect on the chronotype, after controlling for the covariate parental education. This study highlights the need for further research on the chronotype of the morning children in order to regulate their behaviour. The data that were obtained raise questions that have not been yet considered in the literature in the area of education and infant development.

## 1. Introduction

Experimental studies in the field of chronobiology have allowed us to understand the relationship between the 24-hour cycle and the behaviour and performance of the subjects [1,2,3,4]. In the case of human beings, specifically with regard to the sleep rhythm (one of the biological rhythms that occurs in a circadian way), the most recent studies have examined the preference of the day type, which is biologically predetermined [5]. This preference has been analysed regarding normative and pathological cases as the daytime type and biological traits will have an impact on personality disorders, eating behaviours and endocrine disorders in the shift work context [6,7,8]. Thus, it is important to analyse the perspective of the moderating effect that the chronotype has on human behaviour, especially for children and adolescents [9]. Concerning the young population, specifically the school population, the chronotype may influence their academic achievement, their mood and their psychomotor behaviour. The psychomotor behaviour is addressed as psychomotor agitation as it concerns the level of psychomotor functioning in different periods of the day. We have cognitive fluctuations, as well motor and mood differences during the day. The sleep habits of children explain a part of those differences.

The chronotype is identified according to three types or preferences (which are biologically determined): morningness, intermediate and eveningness. Morningness indicates that the individuals prefer to wake up earlier and perform activities earlier when they are compared with the evening persons. Thus, the acrophase of the morning individuals will occur earlier than the acrophase will for the evening group. The acrophase refers to the optimal period of the day concerning the occurrence of cognitive arousal. Therefore, the morning individuals will benefit from cognitive and psychical activities in the morning period, decreasing their level of performance during the day, especially in the afternoon period. On the contrary, the evening subjects tend to display their best performance and have the most energy during the afternoon (and evening hours). However, the evening individuals have shorter periods of sleep when they are compared with the morning cases. This has particular impact on adolescents that experience more problems during activities if they are scheduled during the early morning [9]. It is expected that sleepiness and low academic performance (and motivation) will be found in the adolescent population. Normally, the intermediate group includes the individuals that are positioned between the morning and evening types. The literature addresses the intermediate individuals as the more common type, with them having fewer constraints deriving from the early or late school or work schedules.

The chronotype impacts the brain activation and modulation [10]. In fact, the chronotype is involved in the neuroplasticity differences among human beings and their cognitive functions [10]. Their schedules will be disruptive if the different chronotypes are conducting the same activities during the same periods. According to Schlarb et al. [10], the eveningness type is normally associated with sleep problems and negative functioning concerning the activities which occur during the day. Morningness is mostly correlated to less impulsivity and a more balanced mood. The occurrence of emotion regulation is strongly associated with the morningness/eveningness types. It should be noticed that aggressive behaviour, especially in children and adolescents, is also explained by the chronotype effects. The daytime behaviours are differently perceived—such as struggling with activities and sleepiness symptoms—by the evening individuals when they are compared with the morning types. Schlarb et al. [11] attested that this evidence would explain the correlation between eveningness and aggressive behaviour.

Regarding aggressive behaviour, we can outline the relationship between the oppositional behaviours in the school population and eveningness [12]. Oppositional behaviour is more completely defined as oppositional defiant behaviour. Here, the main focus is the disruptive behaviour of the young subjects that may affect their speed, learning, concentration and their performance of optimal activity when they are in school and home environments. Regarding oppositional behaviour, it means the psychomotor agitation concerning the brain excitation that is involved in specific situations which are classified as an ‘opposition’ to the norm, mostly in the classroom. That excitation leads to mental tension and irritability. These physiological symptoms will not favour the attainment of healthy and normative academic results, nor will they lead to a positive awakeness state. Agitation is linked to hyperactive episodes that will result in distraction during the performance of academic tasks and evaluation periods. Fewer hours of sleep or late bedtime habits will increase the psychomotor agitation. The individuals with psychomotor agitation are likely to be those that are in the group of oppositional behaviours. However, very little research has been conducted on the psychomotor agitation of children with no comorbidities. Moreover, it is not completely understood in what way it is linked to the chronotype. On the contrary, psychomotor agitation and sleep deprivation or sleep-negative episodes in the patients who are suffering from several disorders are well documented [13,14,15]. Focusing on the chronotype and motor functioning, more recent studies [12,16] revealed that, on the one hand, individuals are tending to be more evening type due to a technology dependence and the occurrence of higher motor activity during technology use, while on the other hand, children have more attention deficits and hyperactivity during the evening (and also, this is true for the intermediate type). It is recurrent in the literature that eveningness is also linked to psychomotor agitation. This agitation is part of the characterisation of the oppositional behaviour, and it is internally related to sleep disturbances and inattention [17,18].

The objective of this study is to identify the relationship between the chronotypes, academic achievement and the behaviours in the classroom. It also aims to understand how these events (achievements and behaviours) are distributed according to different times, due to the chronotype of 1st cycle education children. The children’s learning process has to be understood in the light of the evidence. In addition to this evidence, the chronotype, among other factors that are commonly known as age, gender and socioeconomic status, constitute the moderating factors [4,19,20].

The literature has demonstrated how the chronotype can cause the changes that are observed in school performance, during learning activities at school and at home and/or in the school assessment phase [1,21,22,23,24].

The studies that have been reviewed in the area of child development, with a special contribution from the fields of chronobiology and chronopsychology, suggest that there are worrying indicators for one of the school populations that is determined according to chronotype: the evening group. The literature indicates that there is a relationship to be explored between this population and their classroom behaviour in terms of psychomotor irritability and the disruption behaviours that occur at specific times of the day. Considering the non-clinical children, this population lacks studies as to the impact of their chronotype on their behaviour and academic performance. This study is outlined as a detailed developmental perspective through the study of two groups of subjects (morning and evening) that will allow us to understand whether specific sleep habits are directly moderating the oppositional behaviours in the classroom. It was also assumed that the two populations, which are determined by chronotype, will differ significantly in their academic performance, with there being an advantage for the morning group.

Additionally, under this hypothesis, it should be noted that the morning group will have better attention levels and less psychomotor agitation when they are compared with those of the evening one. Furthermore, the sleep habits of the children will be characterised, and determining their chronotype and the variation within this will hypothetically be determined by their age. Regarding to this, the research design follows four hypotheses: there are significant differences between the morning and evening types regarding their performance of oppositional behaviours in the classroom. The Conners Scale—which was a shortened version that was presented to the teachers—was used, specifically focused on the oppositional classroom behaviour subscale: (1) There are significant differences between the morning and evening types in terms of their academic performance. To study these differences, the school grades were studied on two occasions; (2 The evening children show more frequent inattention and psychomotor agitation behaviours when they are compared with the morning children. The disruptive behaviours were examined based on the Conners Scale; (3) The chronotype varies depending on the child’s age (4).

## 2. Materials and Methods

### 2.1. Participants

The sample of this study consists of 140 children who were aged between 6 and 11 years, wherein 53 (37.9%) were male and 87 (62.1%) were female. The questionnaires were mostly answered by the mother (N = 116, 82.5%), by the father (N = 11, 8.3%) and by another person (N = 5, 3.8%). The results showed that 116 mothers knew their child better in terms of their sleep behaviours (87.9%). The respondents were aged between 15 and 65 years, with the most represented age group being between 31 and 45 years (N = 79, 58.1%), with some of them having completed the 12th grade (43.6%) of schooling.

Regarding the school year, most of the children attended the 3rd grade (N = 44, 31.9%), which was followed by the 1st grade (N = 42, 30.4%), the 2nd grade (N = 33, 23.9%) and the 4th grade (N = 19, 13.8%); the household consisted of at least 2 children (N = 44, 43.6%) and 1 teenager (N = 26, 60.5%). However, not all of the children and teenagers in the households have the same biological parents (N = 42, 38.9%).

### 2.2. Instruments

The assessment of the children’s chronotype—the chronotype questionnaire that was used for the children was a Portuguese version that was validated by Couto et al. [25,26]. The original version is the Children’s Chronotype Questionnaire (CCTQ) by Werner et al. [24]. To determine each child’s chronotype, the study used the Morningness/Eveningness subscale (M/E).

This instrument, in its full version, consists of 27 items, which were divided into three scales, which are the midpoint of sleep (MSF), the morning and the evening (M/E) and, finally, the scale of the chronotype type (CT). It can be administered to the children who are between 4 and 11 years of age, in which case it is filled in by their parents [25,26]. We used the M/E scale.

The M/E scale (Morningness/Eveningness) indicates the child’s circadian preference, which was obtained through the sum of the scores of the questions 17 to 26. The points can vary between 10 (extreme morningness) and 49 (extreme eveningness). In the validated Portuguese version, the morning-type children had scores that were equal to or lower than 23, the intermediate-type children had scores that were between 24 and 32, and finally, the evening-type children had scores that were equal to or greater than 33. The internal consistency of the morning/eveningness subscale is 0.711 (10 items) in the validated Portuguese version [25,26]. And the statistical procedure for computation of scores was used as according to Roenneberg et al. [27]. However, the cut-off values of this scale may vary, as was found in the use of the same Portuguese version of the instrument in one of our previous studies of a Portuguese population, but which had a differentiating condition: emigrants (in Luxembourg). In this study, Figueiredo et al. [28] noted that the cut-off point indicated the lower scores which characterised the chronotypes, with there being greater differences in the intermediate and evening cases.

The CT scale was obtained through the responses that were given for question 27, in which the person who completes the questionnaire read a short text about the types of chronotype and marked the option that most resembled their child. The scores ranged from 1 (no doubt that they are a morning type) to 5 (no doubt that they are an evening type).

The child behaviour assessment—the Portuguese version of the Conners Teacher Rating Scale—was used, which is a reduced version of the Revised Conners Teacher Rating Scale, which was developed by Conners [29]. The Portuguese version appeared in 2003, which was adapted by Rodrigues [30].

This instrument consists of 28 items that measure the specific behavioural problems. The teacher assesses each behaviour on a Likert-type scale—from 0 to 3—depending on its frequency, where 0 corresponds to “Never” and 3 corresponds to “Very frequent”. The score is the sum of all of the items per subscale (the N of the items varied in each subscale). The factor analysis resulted in 4 subscales which were identified as: opposition problems, inattention/cognitive problems, excessive motor activity problems (psychomotor agitation and impulsivity) and, finally, the Attention Deficit and Hyperactivity Disorder Index (ADHD) [19].

These scales grouped the items, and their scores are as follows: the opposition scale (items 2, 6, 10, 15 and 20); the inattention/cognitive problems scale (4, 8, 13, 18 and 22); the motor agitation scale (3, 7, 11, 17, 21, 24 and 27); the ADHD index scale (1, 5, 9, 12, 14, 16, 19, 23, 25, 26, 27 and 28). In this study, the subscales with intentional restrictions were used. Considering the starting hypotheses, not all of the Conners items could be included. The selected items informed us according to the level of impulsivity and agitation, not according to the level of hyperactivity, which was not our focus and was not related to the sample nor to the goals’ study. Thus, these are the items and subscales that were used in this study and in the examination of the intended variables: oppositional behaviour (items 5, 15 and 23); inattention problems (items 1, 4, 8, 13, 14, 16, 19, 22 and 25); excessive motor activity or psychomotor agitation (items 2, 9, 12 and 17). The ADHD scale was not considered for the study.

### 2.3. Research Design

This is a cross-sectional study, and as such, the sample was well defined and it was evaluated at a single moment. The cross-sectional design of it also allowed us to confirm the existence of a relationship between the variables. It was also based on the observational and comparative method since the independent variables were not controlled and it was still possible to compare the differences between the morning and evening groups. Statistical methods were used, due to the fact that the research used statistical tools, thus allowing for us to make a detailed description of the sample in an organised manner.

Self-completion instruments were used (the children’s chronotype questionnaire—QCTC and the Conners Scale for the teachers—short version), and these were answered by the parents and teachers in the schools in Portugal in 2021. The reliability and validity of the self-completion questionnaires, including the QCTC, have been confirmed by several studies [15,20,21]. In short, this study resorted to the observational, statistical, comparative and cross-sectional methods. It followed a quantitative approach that is based on exploratory and descriptive research.

After the project’s approval by the Ethics Committee of Universidade Autónoma de Lisboa, Portugal in March 2021 and with the authorisation of the Directorate-General for Education (DGE) of the Ministry of Education and Science to use the questionnaire instruments in a school environment, informed consent was obtained from the following stakeholders: the School Group Directorate, the parents and the teachers. Then, the battery of instruments were used at different times.

First, the chronotype questionnaire [25] was given to the parents (who gave their prior consent to participate in the study). The questionnaires were completed between March and April 2021. Second, the Conners Scale [30] was completed by the teacher that was responsible for each class during a period of approximately two weeks. Finally, the student grades regarding the Portuguese and Mathematics subjects were collected. This information on the school grades was provided by the educational establishment in which the study took place. The gathering of the academic performance information took place in two stages: in February and July 2021.

All of the research procedures were approved by the National Data Protection Commission and by the Ethics Committee of Universidade Autónoma de Lisboa.

The study procedures involving human participants were in accordance with the ethical standards of the institutional research committee and with the 1964 Helsinki Declaration and its later amendments or comparable ethical standards.

The processing and statistical analysis of the data were carried out using the SPSS (Statistical Package for Social Sciences) software, version 27 (Chicago, IL, USA).

### 2.4. Statistical Analysis

A descriptive analysis of the data was carried out, involving frequency tables, with the inclusion of the absolute frequency and relative frequency. Location measures were used, such as the central tendency, mean and non-central tendency, percentiles, as well as measures of dispersion, such as standard deviation. Through inferential analysis, techniques were used to measure the internal consistency of the new variables, using Cronbach’s Alpha, and the research hypotheses were tested with a repeated series of multivariate analyses of variance. MANOVA was the test that was used to explore and compare the groups of chronotypes concerning their oppositional behaviours. With the MANCOVA and ANCOVA, we added the covariates such as the children’s ages and their parents’ education levels and ages to understand the existence of the moderating effect of the chronotype in the results regarding the disruptive behaviours.

The normality of the means of the groups was verified with the Levene test. When there was no normal distribution in the samples, the Mann–Whitney U test was performed to analyse the differences between the groups considering the defined dependent variables. Thus, it was possible to test all of the hypotheses with different statistical tests, taking into account the nature of the distribution of the variables that were involved in each hypothesis. The significance level for rejecting the null hypothesis was set at (α) ≤ 0.05. For the evaluation of the chronotype, the treatment and analysis of the results were carried out based on the statistical tests that are in the original version [24].

## 3. Results

### 3.1. Reliability of the Measures

Children’s chronotype. The processing, analysis and score attribution of the results were carried out based on the statistical tests that are in the original version [24] and based on a repeated series of reliability analyses to ensure the confirmation of the instruments’ validity and the identification of the three chronotypes. It is important to realise that different percentiles were used in the previous versions and in the same population (that is of Portuguese nationality). In this way, the repeated analyses were checked with the calculation of the percentiles after computing the values in all of the items of the chronotype evaluation scale—Question 17-Question 26. Some of the items had a reverse score (a = one in one question may be a = five in another question of the same scale, as observed by Werner) [24]. Questions 17, 18, 24 and 25 presented a reverse score and they were calculated and recorded according to the following interpretation of the answers.

Thus, it was found that the percentiles ranged between 23 and 43 points, which was considered adequate. The M/E scale had an acceptable internal consistency (Cronbach’s Alpha = 0.62; 10 items) with a mean value of 29.4 points (SD = 4.9). These results indicate that there was a level of confidence that was slightly lower than the result which was obtained in one of the previous Portuguese versions (0.71) [20] and significantly lower than the indicator that is in the original version (0.81) [24]. Regarding the corrected item-scale correlation, most of the coefficients were above *r* = 0.30.

According to Couto’s cut-off values [25,26], two age groups that were between 4–7 years and 8–11 years were considered. Three different categories were used for the computation processes. The results show that 47.8% of the children belonged to the intermediate type, which, when they were placed in order of frequency, was followed by the morning type (27.8%) and finally, by the evening type (24.3%).

In order to identify the population according to the chronotype, the percentiles were calculated, as they are the statistical test that is used by the authors of the instrument in the original version [24] and also by subsequent authors in studies such as the one which calculated the validation of the Portuguese population [25,26]. Thus, the percentiles were calculated after characterising the age groups (4–7 years/8–11 years) of the sample.

The P10, P25, P75 and P90 percentiles were considered as the ‘cut-off points’ for the effective identification of the three chronotypes, and firstly, we refined their specificities as they were presented as follows: percentiles 10 (“extreme morning”), 25 (“moderate morning”), 50 (intermediate type), 75 (moderate evening type) and 90 (extreme evening type). The calculation of the cut-off points also allowed for the generation of a different score (cut-off point) to distinguish between the two age groups (4–7 years and 8–11 years). Therefore, 47 (28%) parents of the children from the first group (4–7 years) and 63 tutors (29.1%) from the second group (8–11 years) answered the questionnaire. The missing data are grounded in the absence of the answers to specific items that measured the child’s placement on the morningness/eveningness scale. The results revealed that there were much higher scores when they were compared to the three versions that had been used to date and identified in our studies with a Portuguese sample: the score changed a little in the percentiles regarding the two age groups when it was expected that there would be a significant variation in the P10, P25 and P75. Attention should be paid to P25, where the score was similar in both of the age groups, with there being an unexpected increase regarding the younger group (see Table 1).

This result is a preliminary indicator that the children in this sample belong mostly to the evening type and, above all, with there being less of a difference between them in terms of the chronotypes. From the studies that were carried out with the same instrument and with the samples of the same age group, but in different geographic regions, it was noted that the school populations showed variations according to the region in which they were evaluated. This trend, i.e., there being more evening than morning types, has already been seen in our previous studies of 2018 and 2019, especially in the percentiles 75 and 90 [17] (see Table 2).

The analysis of the percentiles indicates that most of the children are of the intermediate type, and they are distributed very similarly with regard to the morning and evening types (see Table 1). The chronotype scale (Q27) confirmed that 17 (13.4%) people who completed the questionnaire classified the children as being “without a doubt of the morning type”, 19 (15.0%) were the “more morning than evening” type, 40 (31.5%) were the “neither morning nor evening” type, 20 (15.7%) were the “more evening than morning” type, and 20 (15.7%) were classified as being “without a doubt of the evening type”. The missing data refer to the parents who did not answer these items. Those items address the sleep habits and awakeness of their children. Parents have difficulty filling out sleep questionnaires when they become aware of their children’s own schedules. This is one of the problems that we encounter when we distribute this type of questionnaire. The parents do not want to fill them in so as not to reveal their children’s sleep habits (see Table 3).

It should be noted that Q27 was not equivalent to the chronotype characterisation as it was measured by the percentiles and the given age distribution. It was based on the parents’ report of the concepts such as the morning/evening type only.

Classroom behaviour. The internal consistency that was obtained for the three Conners subscales (for the teachers) showed good values (Cronbach’s alpha: ≥0.80) which were only slightly below the indices of the original version (≥0.90, from Conners in 1997). In the Conners Scale, the tendency of the responses to the questions were categorised according to the frequency of the behaviour that was performed by the child, in which “0-Never”; “1-A Little”; “2-Often”; “3-Very Often”.

It was found that, on average, the frequency of the behaviour was mostly categorised in the “A little” category (M = 0.56–1.37), and the rest were placed in the category: “Never manifest” (M = 0.18–0.53). Accordingly, it was decided that we would select the questions with the average answers, “A little” (0.56–1.37), so that the four subscales could be coded. As mentioned in the Instruments section, the following items were considered, excluding the others: oppositional behaviour (items 5, 15 and 23); inattention problems (items 1, 4, 8, 13, 14, 16, 19, 22 and 25); excessive motor activity or psychomotor agitation (items 2, 9, 12 and 17). The ADHD scale was not considered in the study.

Once the items and subscales were selected, the internal consistency that was obtained for the Oppositional Behaviours subscale (0.84), the Inattention Problems subscale (0.93) and for the Excessive Motor Activity subscale (0.92) was detailed. To confirm whether the instruments’ items measured the same construct, the correlation matrix test between the items was performed, in which the corrected correlation coefficients presented values of *r* > 0.595 for the oppositional behaviours, where *r* = 0.482 was the lowest value for the inattention problems and, finally, in the excessive motor activity subscale, the values that were shown were calculated as *r* > 0.710. The relationship between the items ranged from moderate to strong.

### 3.2. Hypothesis Testing and Comparison of Groups

**Hypothesis** **1.** *There are significant differences between the morning and evening types, which are attested by the result of the Conners Classroom Oppositional Behaviours Subscale Reduced Version.*

**Figure 1:**
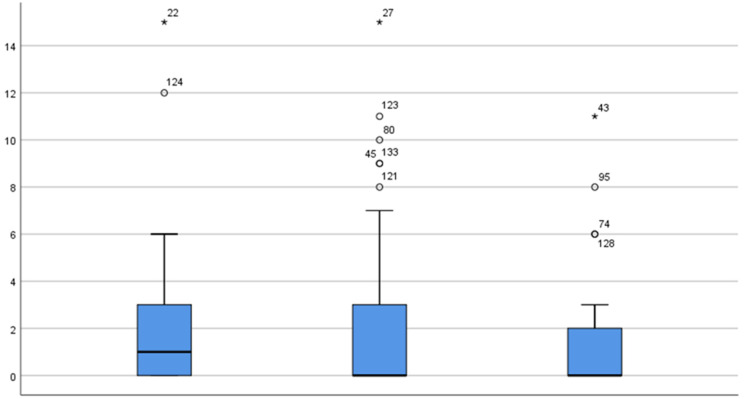
Stem and leaf analysis of the chronotypes * and the oppositional behaviours. Morning, intermediate and evening types (in the order of left to right in the figure) are distributed in the Y axis according to the oppositional behaviours values in the X axis (punctuations are from the scale of the oppositional behaviour).

Oppositional behaviour in the classroom is usually defined as a combination of the disruptive behaviour towards their teacher and their peers. A multivariate analysis of variance (MANOVA) with the groups according to the chronotype as the independent variable indicated the differences between the morning and evening groups regarding the manifestation of oppositional behaviours (dependent variable): *F*(2.110) = 3.156 *p* < 0.05. The effect’s size was average according to Cohen’s parameters (η^2^ = 0.054). The analysis of the homogeneity of variance with the Levene test had been previously carried out and the feasibility of the statistical tests was demonstrated, taking into account the confirmed homogeneity of the results (*p* > 0.05). The post hoc analyses revealed that there were significant differences between the morning and intermediate-type children, with there being a loss for the morning-type children as they presented higher means (M = 3.15) of the frequency of the oppositional behaviours. On the other hand, the intermediate and evening groups have very similar means with there being no significant difference at the *p* level (1.91 and 1.70, respectively). Considering the groups’ means for oppositional subscale: for the morning children, M = 59 (S.D. 1.06), for the evening children, M = 43.1 (S.D. 0.8) and for the intermediate ones, M = 44.2 (S.D. 0.7) (see Figure 1).

In order to control the effects of morningness/eveningness on the oppositional behaviour of the children, the age of the children was included as covariate in the multivariate analysis. Regarding the homogeneity test, the Box’s M test showed a value of 2.096 for the covariance matrices. The objective was to examine whether the level of *p* increased, after controlling for age, the variable of M/E, which did not occur. The regression parameters homogeneity test was used to see whether the M/E could be controlled by the same covariate, and the interaction model did not reveal any significant effects that substantiated the MANCOVA.

**Hypothesis** **2.** *There are significant differences between the morning and evening types in terms of their academic performance.*

The results of the testing of this hypothesis are as follows. Their academic performance was analysed through the school grades: 2 (“insufficient”); 3 (“sufficient”); 4 (“Good”); 5 (“Very good”); (-) (“Does not attend and/or others”). In the 1st semester, the morning and evening children showed very similar grades in the Portuguese subject (morning: 3.45; evening: 3.48). In the 2nd semester, the grades rose both for the morning (mean grade 3.57—sufficient) and for the evening (3.63—sufficient tending to good) types. Regarding the grades in the Mathematics class, the morning-type children had a mean grade of sufficient (3.56), while the evening-type children also obtained a mean grade of sufficient, but with them having better results (3.74). Table 4 shows more results.

The multivariate analysis of variance series did not show significant differences between the groups in terms of their school performance which was measured according to the grades that are described above. In order to confirm the effect of the covariates on the results, the regression parameters homogeneity test was first performed to ascertain whether significant interactions occurred in the interaction model between the variables of M/E and gender, the date of the completion of the test and the parents’ age. The parents’ age normally had a variably moderating effect on the academic achievement of the children. The ages of the families and tutors will influence the parental investment in the education of their children. However, the parameters homogeneity test did not validate the need to use a MANCOVA test.

**Hypothesis** **3.** *The evening-type children have more frequent inattention behaviours and psychomotor agitation when they are compared to the morning-type children.*

The results of the testing of this hypothesis are as follows. The multivariate analysis of variance (we compared the effect of the morning type on the motor agitation/excessive behaviour or motor intensity in the classroom) did not show a statistically significant effect of the chronotype on their levels of inattention/inattention and psychomotor agitation. Afterwards, the regression parameters homogeneity test was carried out to see whether the absence of the effect of M/E on behaviour remained after controlling for the covariates of gender and age. The regression parameter test scores ensured (*p* > 0.05) that the performance of the covariate analysis was possible by confirming the interaction effect model that combined the child’s M/E and gender to analyse their motor excessive behaviour. However, it was not possible to analyse the inattention/cognitive behaviour. Morningness/eveningness appeared to have a controlled *p* effect—F(1.71) = 8.492, *p* = 0.005) after examining the influence that it had on the gender covariate. The effect size was 1.07, which is a low effect according to the Cohen’s d standard. The interaction model did not have a significant effect to justify carrying out a MANCOVA test with other covariates, such as children’s age, for both of the behaviours.

Thus, through observing the means after controlling the M/E variable, the morning type children had higher scores than the evening ones do (and the intermediate type) with regard to the problems of psychomotor agitation and motor excessiveness in the classroom. In short, the differences were only found between the groups regarding motor agitation behaviour and not classroom inattention.

**Hypothesis** **4.** *The chronotype varies depending on the child’s age.*

Result: After conducting a univariate analysis of variance, the differences between the groups did not diverge significantly depending on their age. After having verified the assumptions through the homogeneity test of the regression parameters in order to perform the ANCOVA test, only the covariate “year of schooling” of the parents was seen to moderate the predictive influence of the variable of the children’s ages on the chronotype. Other covariates (for example: gender, time of questionnaire completion and extracurricular activities) were introduced in the model, but only the parents’ schooling was significant in the interaction effects model. In other words, the age groups varied significantly in their determination of the chronotype: *F*(1.11) = 32.323, *p* = 0.000. The effect size was increased according to Cohen’s parameters (η^2^ = 0.0746).

A chi-square analysis was conducted to understand the distribution of the parents according to their educational level and the daytime type of their children. It was found that the parents with more schooling were more like to have a morning-type child.

Taking into account only the average age of the children, at 6 years of age, there is a predominance of the intermediate type (60.9%), at the age of seven, the majority of them correspond to the evening type (47.8%), and there is no effect of age in the intermediate type (34.8%); at the age of 8 years, the majority are confirmed to be in the intermediate type (51.9%), which is followed in terms of frequency by the morning type (25.9%) and the evening type (22.2%); while at the age of 9 years, the majority of them correspond to the intermediate type (57.7%). Finally, at the age of 10 years, most of the children correspond to the morning type (77.8%). As for their sex, the females are mostly represented by the intermediate type (54.9%), while in the male sex, there is an equal prevalence both in the morning type (36.4%) and in the intermediate type (36.4%), with these chronotypes being more expressive in the sample.

In short, the morning children had the worst and highest rate of oppositional behaviours when they were compared, significantly, with the individuals of the intermediate type (no morning or evening preference); there were no differences between the subjects regarding their school performance during the two semesters (covering all of the school periods) according to the chronotype effect, even with the inclusion of the tested covariates; the chronotype influenced the students’ motor agitation and their performance of excessive movement in the classroom, and this affected the morning type more, but only when the covariate of the child’s gender (the M/E) was controlled. In other words, the chronotype was directly affected by the child’s gender, which changed the result regarding their impulsive behaviour. However, there were no differences between the groups regarding their inattention behaviour, and age had an effect on the chronotype after controlling the covariate of parental education.

## 4. Discussion

This study examined the diurnal type of 140 Portuguese primary school children in a specific experiment to understand whether there is a relationship between their chronotypes (daytime preferences for certain activities and sleep behaviours), academic performance, oppositional behaviour and psychomotor agitation in the classroom and if there were differences among these factors according to their age. There were the presuppositions of the tested hypotheses: with the morning children presenting higher risk behaviours when they were compared to the evening children and the children who were classified as the intermediate type. The morning-type children are more undisciplined and show a greater frequency of oppositional behaviours and psychomotor agitation. In terms of their school performance, the hypothesis of the supposition of the differences that were determined according to the three chronotypes was not confirmed. The multivariate analysis of variance allowed for the adding of the verification of interaction models (regression parameters) that confirmed the effect of the covariates such as parental education on the results. The study hypotheses are discussed considering the results that were obtained. All of the analyses of variance were previously subjected to a verification of the normality of the sample distribution to guarantee the principles of the analysis of variance and homogeneity tests.

With regard to the first part of the results, the validity of the two questionnaires ranged from acceptable to excellent (the chronotype questionnaire had the lowest alpha value). The reliability measures followed the steps of the statistical procedures in accordance with the norm of the original versions of the instruments.

Thus, the percentiles were also used on a large scale to confirm the chronotype, and it is important to start the discussion at this point. The Portuguese children in the sample are mostly of the evening type and, more commonly, of the intermediate type. Usually the school-age children are mostly of the morning type, but this was not seen in this study. Even after the multivariate analysis was performed to examine the influence of age on the chronotype, only after controlling the gender variable was it possible to obtain the differences between the age groups with a high effect size. Gender may have a more significant influence than age does, which is in line with a part of the results of the study by Gaina et al. [31]. In this study, the female sex was, in a young sample, more of the evening type than they were of the morning type, but the sleep and wakefulness behaviours were more inadequate in the evening population regarding their age and gender.

In fact, the percentile results showed that there were similar and high scores that were used to distinguish between the morning and evening types (P25 and P75), which conflicts with most of the studies that attest to there being more distant and distinct cut-off points for the morning and evening populations in 6-to-11-year-old children [32]. Probably, the change to the evening type that occurs at around puberty [33] happened earlier in the more recent population, given the date of the studies that were carried out with the children and adolescents. The results of this study make a distinct contribution in the area of child development with regard to the specific development of chronotypes in the age group of 4–11 years. Younger children would be expected to be more of the morning type than the evening type. All of the children had scores that were close to those of the evening type, so the more advanced morning cut-off point (similar to the cut-off point for the 75 percentile—eveningness) suggests that children have more difficulty waking up and being awake (as well as going to bed early, without parental imposition occurring). The cut-off points that are seen in the chronotype scale allowed for the understanding that the development of sleep and wakefulness habits may be more altered nowadays in children, such as by keeping them cognitively awake at later times and with them having later acrophases regarding their performance. These types of indicators also suggest that the classroom behaviours are less normative for the morning children than it is normally expected to be. Sleep behaviours are moderated by age, gender and the type of habits that are learned at home (this is the parents’ responsibility). Even considering that adolescents have a tendency (biologically determined) to wake up later than young children do, they will be affected by the regular school schedules that demand that they begin classes and activities earlier [1,2,3,4,5]. As previously maintained, the morning types perform better in physical and cognitive activities during the morning period, with a decrease in their optimal condition occurring later in the day. The evening subjects prefer to accomplish tasks in the afternoon period, and they can attain better grades in the afternoon period. Sleepiness will be a disadvantage for the adolescent population [9,10].

Previous results may partially explain the confirmation of hypothesis 1, as there are significant differences between the subjects according to their chronotype, with there being a disadvantage for the morning ones regarding the performance of oppositional behaviours in the classroom. The morning students have the greatest oppositional behaviour, therefore they are undisciplined. The morning schoolchildren had cut-off points that were very close to the scores which were assigned to the evening children, so there may be a more evening trend that is associated with the performance of oppositional behaviours in the classroom. These behaviours are easily related to their causes which are explained by Martínez-Lozano [33]; the evening children, precisely in this age range, tend to present more irritability behaviours due to them having higher cortisol levels (less positive metabolic changes in child development) and them falling asleep later, this explains them having a later acrophase. The child’s development is thus affected from school age onwards, with them experiencing social jetlag and sleep deprivation that will generate drowsiness and behavioural problems in the classroom. Indeed, more recent studies point to there being more eveningness in school-age children [33,34,35,36], and when examining the children who were identified as evening ones, they noticed that they had greater health problems, and they were more undisciplined. Still, while the evening children have been studied recently, this does not mean that these studies corroborate the higher percentage of morningness in this age group, nor the positive relationship between eveningness and good behaviour, rather this is quite the contrary. The extensive literature review that has been carried out allows us to find a discrepancy between the chronotype results of this sample and the results of the studies on child sleep and wakefulness. On the other hand, the evening type, regardless of their age, is regularly associated with behavioural problems [36]. One of the problems lies in the poor empirical analysis of the chronotype of school-age children, especially in the relationship between their evening life and classroom behaviour. The need to verify and replicate the cut-off points of the questionnaires considering the different populations of the children in different geographic contexts is also very important. Social and school hours can vary considerably between countries and regions, which generates this type of discrepancy in the results.

With regard to hypothesis 2, it was not confirmed because there were no significant differences between the morning and evening types in terms of their academic performances, specifically regarding their grades in Portuguese and in Mathematics classes, as these are two very different subjects. These results are not surprising since the cut-off difference (in percentiles) was so low for the morning/evening variable. Accordingly, it was expected that the differences regarding performance would not be significant between groups. On the other hand, our data are supported by the study by Roeser et al. [37] and by Arrona-Palacios et al. [38] because the chronotype was not directly related to the children’s performance, but rather to their personality traits. Randler et al. [38] also found that the chronotype did not influence the performance on math tests, although it had great impact on the affective and emotional domains.

As for hypothesis 3, it was rejected because the evening children did not show higher inattention and psychomotor agitation behaviours. On the contrary, the morning children were more restless and had an excessive level of agitation in the classroom when they were compared with the evening group. This result is a new contribution, and it conflicts with studies that have empirically linked the evening type to psychomotor agitation and inattention. Extreme impulsivity is often related to the wrong responses in the go/no-go paradigm and motor control problems [39]. A few studies were found that support our results for this hypothesis [40]. In these studies, there were low or inexistent (significant) differences between the chronotypes regarding the level of inattention and agitation behaviours [41]. The use of the Conners Scale (involving the version for teachers as well as the version for parents) usually focuses on the populations with hyperactivity disorders, autism and other pathologies (even involving sleep pathologies, without considering populations with normative sleep patterns). Fewer normative populations are studied in order to explore the relationship between the children’s psychomotor agitation and impulsivity (non-clinical sample) and sleep. The correlations that were found between the morning type and excessive motor behaviour in the classroom do not seem to agree with the assumption of non-oppositional behaviours in the morning type. The literature points in the opposite direction, which is why it is important to understand the issue of morningness, whose values that are not very different from the evening percentile ones.

The studies that have most often used the instruments to assess children’s chronotypes have associated the evening children with poor general health rates [42]. Overwhelming research has linked the evening children to a poorer school performance, oppositional behaviour and sleep problems, which was not confirmed in this study. However, with the pandemic phase which occurred and the fact that this study took place during the third European wave of COVID-19, this may be a factor that was not controlled, which may enable us to understand the absence of the chronotype differences in the children. The morning and evening differences did not manifest themselves differently than they usually do as there may have been an abrupt change in the sleep behaviour of the children due to the use of screens for online school activities and also because of the complete change in the schedules and habits of families that were staying at home. The family system and dynamics changed during this period, and it is very likely that their sleep routines have been affected and that their chronotypes have been calculated based on the influence of the lockdown period.

This leads to another issue that is related to the need to reassess the current chronotypes of the younger children and the implications of the chronotype changes in child development, specifically with regard to oppositional behaviour, psychomotor agitation and academic performance. Additionally, according to the study by Türkoğlu et al. [43], the lockdown context influenced the most common chronotype, that is, the more evening-type children. On the other hand, their age was a variable that must be taken into account to explain our results that did not confirm the impulsivity and disruptive behaviours of the evening group. Advancing in age (even when they are very young) explains why the morning and evening types were not contrasted as the past literature has established [44].

Finally, the testing of hypothesis number 4 has partially confirmed that age initially did not significantly influence the determination of the chronotype. After controlling the covariate ‘parental education’, the results were modified by this factor. These data are robustly supported by the studies such as the ones by Karan et al. [45] and Haldar et al. [46] because the level of education of the parents is one of the covariates that is believed to determine the chronotype. However, in the study by Haldar et al. [46], the parents having a higher level of education/skills influenced the more evening-type children, as well as sleep routines that are associated with this chronotype. In our study, the parents with higher education qualifications are more likely to have morning children. However, Haldar et al. [46] associated the contextual variables such as living in urban or rural areas, which may certainly have moderated the results which were obtained. Regarding the context as a variable, the period when the tests were conducted must be taken into account: during the third wave of the pandemic in Europe. This context may have modified the data, and this may justify the fact that morning children mainly have parents with high educational qualifications. Teleworking, especially for professionals with higher qualifications and professional positions, affected the routine at home, which may explain the association between the morning hours and their psychomotor agitation behaviours in the classroom (and at home), considering hypothesis 3. There are no studies on this relationship between the parental qualifications and the children’s chronotype, and there, especially, there are none on parental education determining the effect of age on the chronotype. This line of results focusing on age and chronotype can be explained in a first perspective that relates to the pioneering North American research on the prevalence of chronotypes, which considers age as a determining and modifying factor. The chronotype varies according to age but in a decreasing direction according to Fischer et al. [47].

As previously mentioned, the adolescent population was the most worrying given that they have a delay in their phase (they tend to wake up later, which generates disruptive behaviour in this age group regarding the beginning of the school day). Jankowski [48] also examined the variability of the chronotype according to age, with the chronotype tending to increase from puberty onwards and regress again in the elderly phase (increasing and regressing is understood as increasing scores and decreasing percentiles). From another perspective and in another sense of the research that is under the topic of discussion, existing studies have focused mainly on the variability of the chronotype over time (according to age, above all) and they mainly use adolescent and mostly adult populations [49].

Ultimately, there are no sufficiently competitive explanations to enlighten the results that we have obtained, especially considering that the effect of age was moderated by the educational qualifications of the participants’ parents (respondents of the Conners Scale). However, the study by Dimitrov et al. [50] on the effect of the chronotype on psychological well-being must be considered. The authors controlled the age and gender variables, assuming that it is the composition of the sample that defines how the chronotype affects their well-being or not. Sládek et al. [51] also analysed the effect of the chronotype, taking into account factors such as age and gender, but mainly involving the contextual and geographic variables. The context variable reappeared as a moderator and predictor. Currently, studies have focused on an empirical line that equates and explores the relationship of the chronotype with more variables that go beyond age. Werner et al. [24] determined the influence of age on the chronotype, which supported the formula for calculating the percentiles to differentiate the chronotype in school-age children. Age is always the main differentiating factor of the chronotype for determining and validating the instruments. However, age is found to be a less predictive factor than it was expected to be in this study, and the variability of the chronotype is almost absent throughout the different ages of the sample.

## 5. Conclusions

The use of well-established and validated measures for the studied population made it possible to generate an important contribution to the field of understanding child development in the following relationship: chronotype (sleep), classroom behaviour and academic performance. This study provides a unique result, considering the reputable literature about the relationship between the morning children and a high rate of oppositional behaviours, in addition to the fact that these children have a very high eveningness index.

This evidence contradicts the previous studies that support, on the one hand, the positive and proportional relationship between morningness and non-oppositional behaviours (nor significant motor agitation). On the other hand, it suggests the extreme eveningness trend that is seen in school-age children (6–11 years old) and the influence of the parents’ education as an age moderator in defining the chronotype. The nature of the sleep and disruptive behaviours of the tutors themselves may be more indicative than the nature of the sleep in the age groups that we studied. In addition to presenting a contribution that needs to be replicated and deepened in other populations with the same age groups and using the same instruments, this study intends to draw attention to the implications that it has for child development. In addition, context should be a variable that is added in future studies, given the durable nature of the pandemic and the different repercussions that it has had on the populations in each country. The influence of family education on the child’s chronotype (their routines were probably altered by the pandemic for the parents who have professional positions that demand, above all, very altered daily routines) should also be taken into account. The tendency to increase the children’s chronotype is highly likely during the periods of lockdown. The studies in the area of sleep and child development should focus on the impact of morningness on disruptive behaviours, rather than analysing how the chronotype variability is processed over time. From this developmental perspective, it will be possible to monitor the rhythms of the children of different age groups with different family backgrounds based on their needs and routines which are informed by their parents and teachers.

As for the limitations, we need to address the cut-off low (and very similar) values that were found for the morning and evening children which may be affected the results. Additionally, the alpha value which was achieved for the chronotype scale was low, even if it was acceptable in statistical criteria. It must be pointed out that the missing data could have affected some of the results. In further research, the researchers should conduct a more in-depth analysis on the evening-type school-age children. Moreover, we intended to control the social variables, and it would be useful to add multi-level models for a more complete statistical exploration of this.

## Figures and Tables

**Table 1:** Morning/Evening Scale Score for two age groups—parents’ self-report.

		4–7	8–11
N	Valid	47	63
	Missing	12	10
Median		29.09	28.00
Std. Deviation		4.84	4.84
Skewness		0.43	0.58
Kurtosis		0.55	0.23
Std. Error of Skewness		0.35	0.59
Minimum		20	20
Maximum		43	42
Percentiles	10	23.00	23.00
	25	27.00	26.00
	75	33.00	31.00
	90	36.00	35.60

**Table 2:** Sample characterisation according to chronotype.

		Frequency	%	% Valid	% Cumulative
Valid	Morning	32	22.9	27.8	27.8
	Intermediate	55	39.3	47.8	75.7
	Evening	28	20.0	24.3	100.0
	Total	115	82.1	100.0	
Missing		25	17.9		
Total		140	100.0		

**Table 3:** Results of the use of the chronotype scale.

	N	%
Without a doubt of the morning type	17	13.4
More morning than evening	19	15.0
Neither morning nor evening	40	31.5
More evening than morning	20	15.7
Without a doubt of the evening type	20	15.7
Do not know	11	8.7
Total	127	100.0

**Table 4:** Academic performance according to morningness/eveningness.

	Morning	Evening	
	Mean	Mean	*P*
Grade in Portuguese (1st sem)	3.45	3.48	0.91
Grade in Maths (1st sem)	3.56	3.74	0.46
Grade in Portuguese (2nd sem)	3.57	3.63	0.82
Grade in Maths (2nd sem)	3.57	3.52	0.87

## Data Availability

These and other supplementary data that are related to this study’s materials are available in our archives of SPSS and respective syntaxes. Additionally, this work was not preregistered (not applicable). The project’s study was approved by the Ethics Committee of the Universidade Autónoma de Lisboa, Portugal.
